# The Effect of Forward Testing as a Function of Test Occasions and Study Material

**DOI:** 10.3390/bs11090114

**Published:** 2021-08-24

**Authors:** Robin Sohlberg, Fredrik Olsson, Pierre Gander

**Affiliations:** Department of Applied Information Technology, University of Gothenburg, Forskningsgången 6, 417 56 Göteborg, Sweden; sohlberg.robin@gmail.com (R.S.); fredrik.olsson.92@gmail.com (F.O.)

**Keywords:** encoding, face-name pairs, forward testing effect, interim tests, learning strategies, prior list intrusions, recall, transferability

## Abstract

It has long been known that one of the most effective study techniques is to be tested on the to-be-remembered material, a phenomenon known as the testing effect. Recent research has also shown that testing of previous materials promotes the learning of new materials, a phenomenon known as the forward testing effect. In this paper, as of yet unexplored aspects of the forward testing effect related to face-name learning are examined; continuous and initial testing are compared to restudying, the effects of an initial test on subsequent learning, and whether an initial change of domain (change from one topic to another) regarding study material affects the robustness of the effect. An experiment (N = 94) was performed according to a 2 (Material: word pairs/face-name pairs in Block 1) × 3 (Test occasions: Blocks 1–4/Blocks 1 and 4/Block 4) complex between-groups design. The results showed that no difference between testing and repetition could be observed regarding the recall of faces and names. The restudy groups incorrectly recalled more names from previous lists in the last interim test compared to the tested groups, which supports the theory that interim tests reduce proactive interference. The results also suggest that the number of test occasions correlates with the number of incorrect recalls from previous lists. These results, in contrast to previous studies, highlight a potential uncertainty about the forward testing effect linked to the robustness of the phenomenon, the specificity in execution, and generalizability.

## 1. Introduction

In a society that is constantly evolving, the ability to learn new things in effective ways is very valuable. Learning is not only a central part of education and professional endeavours but is essential all throughout life. It is reasonable to assume that the choice of learning strategy affects the process of learning in different ways. Dunlosky et al. [1] discussed 10 study techniques and rated them based on their utility. Their conclusion was that one of the most useful study techniques is—during the actual study period itself—to be tested on the material you want to remember. By performing practice tests, the retention of the material is promoted, and this phenomenon is known as the testing effect. The testing effect was discovered as early as 1909 [2] and has strong empirical support (see [3] and [4] for a review of the literature).

Despite the strong empirical support for the testing effect, there are indications that knowledge about testing as an effective learning strategy in society could potentially be improved. An example of this can be observed in a study by Karpicke et al. [5] where it was investigated to what extent students used self-testing compared to other study techniques. In the study, a question was asked regarding which study technique the participants would prefer if they were to, hypothetically, take a test on a book chapter that they had just read. Participants were given the following options: (A) read the chapter again (or parts of it), (B) try to recall the material from the chapter (with no opportunity to study the material again), or (C) use any other study technique. The results showed that 57% answered A (read the chapter again), 21% answered C (other technique) and only 18% answered B (try to recall the material from the chapter). When it comes to long-term retention of the material, alternative B is one of the most effective study techniques, especially in comparison with repetition [6], yet a large majority chose alternative A. Although this is a single example, it still emphasizes the importance of disseminating knowledge about, exploring, as well as implementing effective learning strategies. Effective learning strategies can potentially help many people to learn more, in less time, as well as retain more of what they learned in the long run. Optimized knowledge acquisition can be of great benefit to the individual as well as to society as a whole.

A new discovery made in recent years is that testing of previously studied material can also potentiate learning of subsequent materials. This phenomenon has been named the forward testing effect (FTE). A common experimental approach when it comes to examining the forward testing effect is to divide the material to be remembered into several separate study blocks (for some examples of this, see [7,8,9]). The participants are then divided into at least two groups; a test group and a control group. The test group is tested on each individual study block, just after they have studied it (this type of test is called an interim test) while the control group either performs a distraction task or restudies the content a second time; the control group is then tested only on the last study block. To see the effects of forward testing, one can look at the results of the interim test for both groups on the last study block. Since both groups take an interim test on the last study block, it can be assumed that the differences between the groups regarding the result on the last block are due to the test group undergoing interim tests in the previous blocks, which leads to FTE.

FTE has been found in a number of different studies, and the evidence suggests that the phenomenon is robust (see [10] for a review of the literature). The effect has been linked to several different types of learning; single item learning in the form of individual words [7,9,11,12,13,14,15,16,17] and pictures [18], face-name pairs [8,9,19] or word pairs [9,20], complex materials in the form of video lectures [21,22,23,24] and text passages [25,26,27], inductive learning in the form of painting styles of artists [28,29], self-regulated learning in the form of word pairs with unlimited study time [9] and in learning through domain changes (switching from one topic to another) with regards to study materials [19].

This paper describes a replication of the classic effect of forward testing through a combination of word pairs and face-name pairs in order to examine how the learning of one material affects the learning of a different material through forward testing. Additionally, the number, and timing, of test occasions are manipulated to see if an initial test can produce a similar effect as being tested continuously.

### 1.1. Theories of the Forward Testing Effect

Although FTE has proven to be a robust phenomenon, there are still uncertainties regarding which mechanisms contribute to the effect. The theories that attempt to explain the mechanisms underlying FTE are several, and mechanisms that operate during either the encoding or recall phase, or both, are believed to contribute to the effect. In addition to the fact that each theory is mainly linked to either the encoding or recall phase, they can also be divided based on whether they are motivation-based or not. Yang et al. [10] mention that it is important to emphasize that most of the theories are not mutually exclusive, that they are in an early stage of development and that few have been subjected to direct testing of their most important predictions. Below are brief descriptions of the eight theories that currently exist.

#### 1.1.1. Reduced Proactive Interference

Szpunar et al. [7] suggested that interim tests induce mental context changes and that this is the main underlying mechanism behind FTE. According to the theory, interim tests make the material associated with both learning and test contexts, which makes it easier to distinguish the materials from each other. These context changes then reduce the build-up of proactive interference, that is, the extent of previously learned objects that interfere with the encoding or recall of subsequently learned objects, which improves the recall of the target objects.

#### 1.1.2. Encoding Reset

Pastötter et al. [12] suggested, similar to Szpunar et al. [7], that mental context changes induced by interim tests could constitute an underlying mechanism behind FTE. Pastötter et al. meant, however, that these context changes could reset the encoding process, which would make the encoding of subsequent information as efficient as the encoding of previous information.

#### 1.1.3. Strategy Shifts in Encoding/Recall

Cho et al. [20] suggested that FTE may be caused by a change in strategy linked to encoding and/or recall. With continuous interim testing within the same domain and with the same test format, participants can become informed about the test format and consequently adapt their encoding or recall strategies to make the encoding/recall of new information more efficient.

#### 1.1.4. Improved Accessibility

Wissman et al. [25] suggested that a possible mechanism behind FTE, when learning text material, could be that an interim test on a previous section improves the memory of the tested information, making the tested information more readily available when reading a subsequent section. Improved availability of previously learned information could increase the understanding of subsequent information. However, Wissman et al. emphasizes that it is still unclear how the learning of separate texts is potentiated (regardless of subject relationship) through improved accessibility if the information could not be compared contrastively.

#### 1.1.5. The Test Expectancy Theory

In a study by Weinstein et al. [11], participants had to study five lists of words. The participants were divided into four groups that were either tested or not, as well as whether or not they were warned ahead of the last block that they would be tested on the last list after studying it, giving the groups the names of Tested, Not-Tested, Warned-Tested, and Warned-Not-Tested. The Warned-Not-Tested group performed better than the Not-Tested group regarding correct word recall and experienced less proactive interference through fewer incorrectly recalled words from previous lists (prior list intrusions). In a follow-up experiment, the participants’ perceived probability that they would be tested on the following list was examined. The results showed that test expectancy increased in the Tested group and decreased in the Not-Tested group. According to Weinstein et al., this suggests that interim testing protects against proactive interference through attention-based processes and/or more efficient encoding as a result of high test expectancy.

Yang et al. [19] found the same pattern, where test expectancy increased in the test groups and decreased in the control groups.

#### 1.1.6. Increased Effort during Encoding or Recall

Two additional theories were proposed by Cho et al. [20] who argued that failed recall in previous interim tests could motivate greater effort toward either encoding or recalling new information. In line with the theory of increased effort during encoding, several studies have shown that failed recall or mistakes made in previous tests can potentiate subsequent encoding [30,31,32].

### 1.2. Previous Studies

#### 1.2.1. Word Lists

Szpunar et al. [7] conducted a FTE study, which is now known as a classic, where the purpose was to investigate whether testing during a study phase protects against the build-up of proactive interference. To investigate this, they developed a new paradigm where participants had to study five lists of words. Participants were informed that, after completing all five lists, they would be taking a final cumulative test on all lists (this was done to maintain the learning engagement across the blocks). The study consisted of four experiments.

Experiments 1A and 1B used the same procedure and materials (except for experiment 1B, which had other words). The participants were divided into two groups; a test group and a non-test group, both of whom had to study five lists of words. After studying each individual list, both groups had to solve math tasks, and then the test group was tested on the list just studied and the non-test group had to complete additional math tasks. Participants were informed that whether or not they would be tested after each list was decided by the computer program (which, in reality, was predetermined). The results from both experiments showed that participants who had not been tested after lists 1–4 recalled significantly fewer words from list 5 on both the initial and cumulative test and had more prior list intrusions on list 5 compared to the test group.

The purpose of experiment 2 was to investigate performance in relation to the occurrence of proactive interference. The participants were divided into five different groups; a test group that was tested immediately after studying each list and four groups that were tested only once on one of the lists 2–5 immediately after they studied the list (one group was tested on list 2, one group was tested on list 3, etc.). The four groups that were not continuously tested were thus exposed to different numbers of untested lists before they were tested. The material was identical to that used in experiment 1A and the procedure was the same as in experiments 1A and 1B. The results of the interim tests showed that as the number of previously untested lists increased, the number of correctly recalled words from a new list decreased and the number of prior list intrusions increased.

In experiment 3, the participants, just as in the previous experiments, were given the task of studying five lists of individual words. Participants were informed that after studying each list, they would first have to solve some math problems and then either be tested, get to restudy, or do another round of math problems. The participants were divided into three groups; a test group, a restudy group and a distraction group. The test group took an interim test (free recall) after studying each list. The restudy group restudied the list again after studying each list (1–4), and then had to take an interim test on list 5. The distraction group had to perform additional math tasks after studying each list (1–4) and then had to take an interim test on list 5. The results showed that the test group recalled approximately twice as many correct words on the interim test of list 5 compared to both the restudy group and the distraction group. The test group also had fewer prior list intrusions on the interim test on list 5 than both other groups. No major difference in prior list intrusions was shown between the restudy group and the distraction group, and thus this result showed a clear effect of forward testing on the test after list 5 as well as a large effect on the reduction of proactive interference. The test group also performed significantly better than the restudy and distraction groups in the cumulative test.

#### 1.2.2. Face-Name Pairs

After the findings from Szpunar et al. [7] a number of studies were conducted that built on the interim test paradigm to examine the learning of paired associates in the form of face-name pairs [8,9,19]. To investigate FTE with face-name pairs, all of these three studies used a similar approach as Szpunar et al., but without a restudy condition. The participants were thus only divided into two groups; a test group and a distraction group that had to study four lists of face-name pairs. During testing, participants had to perform a cued recall test (recall using a clue, in this case the faces without the names) where they would type the name associated with each face. Participants were informed that, after studying each individual list, they would first get to complete math tasks and then have to either solve more math tasks or be tested on the list they had just studied, and that this was determined randomly by the computer program. In reality, the test group was tested after each list and the distraction group was only tested after list 4. They were also informed that they would take a final cumulative test that included all face-name pairs from all lists. The results in all three studies showed that the test group, like the results from Szpunar et al. [7], recalled approximately twice as many correct names on the interim test of the last list (list 4) as the distraction group. In all three studies, the test group also showed significantly less proactive interference (by measuring prior list intrusions) in comparison with the distraction group.

#### 1.2.3. Transferability of the Forward Testing Effect

Yang et al. [19] examined the question of whether the interim testing of studied information in one domain can promote learning and retention of new information in a different domain; in other words, if the effect of forward testing is transferable. To investigate this, three different experiments were performed.

The first experiment examined the transferability of FTE by having participants study three lists of Swahili-English word pairs (Blocks 1–3) followed by a list of face-name pairs in the fourth block. Half of the participants were tested after each list and the other half had to perform a distraction task on the first three blocks and a test only after the fourth block. The results showed that those who were tested on the word pairs in Blocks 1–3 performed better in the test on face-name pairs than those who were not tested, which indicates that FTE is to some extent transferable.

The second experiment examined whether the effect is transferable between images, text and face-profession pairs. This was done with a test group that was tested after each block and a restudy group that got to restudy the first two blocks with a test only after the third and final block. To control for any strategy shifts regarding encoding/recall, the test formats were different in all three blocks. The results of the test in Block 3 revealed a performance difference between the test and restudy group with approximately twice as many correctly recalled professions in the tested group, thus showing further indications of the transferability between domains.

Experiment 3 aimed to investigate whether FTE is transferable between different levels of learning; remembering specific objects (lower level) and inductive learning (higher level). The experiment consisted of four blocks and was performed with a test group and a restudy group. The first three blocks consisted of learning at the lower level in the form of short statements about artists. In the fourth block (with a high level of learning), the participants got to see paintings by different artists. During testing on the fourth block, new paintings by the same artists were shown and the task was to determine which of the artists had painted each painting. The results showed that the test group to a greater extent classified the paintings correctly compared to the restudy group, which indicates transferability of the effect between verbal facts and visual concepts.

In their meta-study, Yang et al. [10] recommended that further research should be carried out to find out to what extent the effect of transferability potentially weakens the effect of forward testing.

#### 1.2.4. Demonstrated Limitations with Forward Testing

Although FTE has largely been shown to promote learning, there is some research indicating that interim tests can have a negative effect on learning. In a study by Finn and Roediger [33], an experimental approach to FTE that proved to be counterproductive was observed when participants who first studied face-name pairs, in a later list, got to study the same face-name pairs again but with additional information in the form of occupations. In Finn and Roediger’s study, the participants were assigned to either a test group or a restudy group and the hypothesis was that those who were tested on face-name pairs before the addition of occupations would remember the occupations better than the restudy group, which did not turn out to be the case. In a cumulative test 24 h later, the test group was able to report more correctly recalled names for each face, but the restudy group reported more correctly recalled occupations in comparison to the test group. Thus, it seems that a negative aspect of FTE includes cases where complementary associations are added later, whereupon this additional information becomes more difficult to remember if one has been tested on parts of the information before. Practical applications should therefore keep this in mind when structuring the information that is to be promoted through forward testing.

### 1.3. The Present Study

Although a great deal of research has been done on the forward testing effect, there are still questions that remain unanswered. The purpose of this study is to investigate two as yet unexplored aspects of FTE as well as whether a modified replication of the face-name pair studies above [8,9,19] with higher ecological validity can reproduce the robust effect previously observed of the phenomenon.

Through this replication with the modification that the group that is not tested gets the chance to study the material again (similar to experiment 3 in [7]) instead of solving additional math tasks, the ecological validity of the experiment should be higher. A study technique where people study a material followed by 90 s of unrelated mathematics does potentially lack ecological validity. It is reasonable to assume that FTE is more practically relevant when compared to another legitimate study technique, in this case repetition (as shown by Karpicke et al. [5], to be the preferred study technique). This will allow a more equal exposure opportunity of the material, and the difference will instead be more refined in the form of exposure technique. In addition to this replication, which (1) examines whether the effect of forward testing can be reproduced with higher ecological validity, the yet unexplored aspects include (2) whether an initial test at the beginning of the experiment can have similar effects on recollection as being tested after each study session, (as the theory of test expectancy states that the expectation of being tested would increase following prior tests and thus potentiate the FTE) and (3) whether the effect of forward testing remains robust when the initial study material differs from the subsequent materials. In addition to seeing what effect these interventions have on learning new materials, the study will also explore what effect the interventions have on the previously observed effect that proactive interference is reduced through interim tests.

If an initial test shows similar effects on learning as being tested after each study opportunity, this insight will potentially be practically significant as fewer tests could be performed during a study session and still give comparable results. If FTE proves to be robust after an initial domain change regarding study material, this may indicate that a test after a study session promotes the learning of the subsequent session, even if the sessions consist of different types of materials. Insights from these two unexplored factors in combination could then potentially be applied in practice by using a standardized test before a study session, which would be feasible on a large scale at all levels of education for those who want to make use of the phenomenon to increase our collective knowledge aggregate.

## 2. Materials and Methods

### 2.1. Participants

Based on the mean value (d = 0.69) of the effect sizes (d between 0.58 and 0.87) found by Yang et al. [19] in relation to the transferability of FTE and the mean value (d = 1.41) of the effect sizes (d between 0.7 and 2.78) found by Szpunar et al. [7], Weinstein et al. [8] and Yang et al. [19] for the general effect of FTE, the sample size for this study was calculated using the software G*Power to amount to at least 94 participants with power = 0.8 and alpha = 0.05.

A convenience sample generated a total of 94 participants (41 women, 50 men, 3 non-binary) aged between 14 and 83 years (M = 31.47, SD = 13.04). The study was shared on Facebook, Reddit.com (/r/Sweden, /r/SampleSize, /r/CogSci, /r/Psychology) as well as through a number of survey exchange activities. In order to reach and recruit a sufficient number of participants, 60 participants were recruited through a Swedish version of the experiment and 34 participants were internationally recruited through an English version of the experiment.

### 2.2. Material

Forty images of female faces were downloaded from https://thispersondoesnotexist.com/ (accessed on 1 June 2021). Regarding the Swedish version of the experiment, 40 female names were taken from a list of the most popular names of newborns in 1998 from svenskanamn.se (1998 was the earliest available year). Regarding the English version of the experiment, 40 female names were retrieved from a similar list on the Social Security Administration website (https://www.ssa.gov/oact/babynames/decades/names1990s.html (accessed on 1 June 2021)). The face pictures and names were paired randomly, and all participants saw the same face-name pair. The order of each face-name pair was randomized and kept constant for all participants during the exposure phases. In the test phases, the order of the face-name pairs for each participant was randomized. Furthermore, 10 English words from 10 semantic categories (one word from each category) were chosen based on the category norms from [34]. The words were translated into both Swahili and Swedish, which formed the word pair for each language version of the experiment (Swahili-English and Swahili-Swedish).

The experiment was programmed in, and performed using, PsyToolkit [35,36].

### 2.3. Design

**Figure 1:**
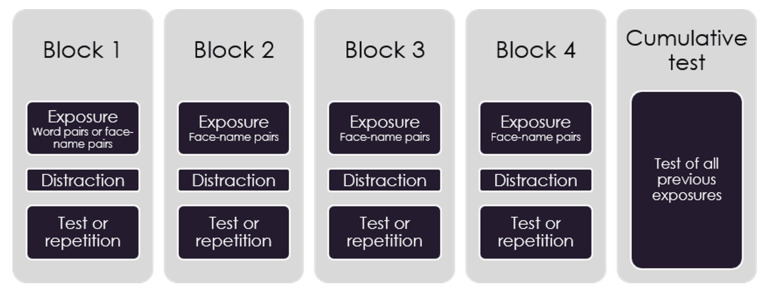
Experimental design.

The experiment was performed using a 2 (Material: word pairs/face-name pairs in Block 1) × 3 (Test Occasions: Blocks 1–4/Block 1 and 4/Block 4) complex between groups design. The participants were randomly assigned to 1 of 6 groups; 16 in Transfer-Test, 15 in Transfer-Restudy, 16 in Transfer-Initial Test, 15 in Classic-Test, 16 in Classic-Restudy, and 16 in Classic-Initial Test. The experiment was initiated with four study blocks, each consisting of an exposure part, a distraction part and a test or repetition part (see Figure 1). The distraction parts consisted of 20 different simple math tasks (e.g., “99 + 2”, “40/20”) per block. After Block 4, the experiment was concluded with a cumulative test involving all prior exposures. The groups that were exposed to word pairs in the first block are called Transfer-Test, Transfer-Restudy and Transfer-Initial Test and the groups that were only exposed to face-name pairs are called Classic-Test, Classic-Restudy and Classic-Initial Test. Furthermore, the timing and frequency of testing in the different groups were varied as follows: Transfer-Test and Classic-Test were tested after each block, Transfer-Restudy and Classic-Restudy were tested only after Block 4, Transfer-Initial Test and Classic-Initial Test were tested after Blocks 1 and 4. All six conditions underwent the cumulative test immediately after Block 4. The two dependent variables used were the number of correctly recalled names and the number of prior list intrusions when tested on the fourth block.

### 2.4. Procedure

**Figure 2:**
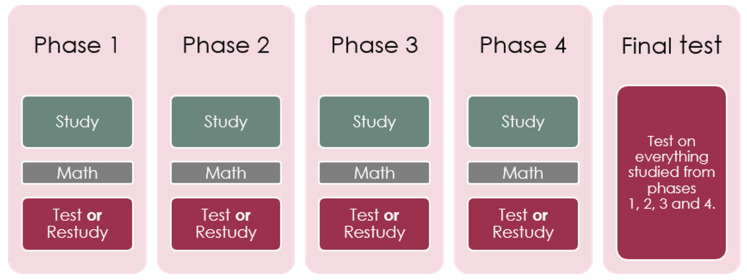
Simplifying diagram which supplemented the information page.

Before starting the experiment, the participants gave their informed consent to participate. At the start of the experiment, the participants were presented with the experimental set-up where they were informed that they would undergo four blocks of three sections each, including what those parts involved, and that they eventually would be tested on everything they studied. Along with these instructions, they were also shown a diagram designed to simplify the understanding of the set-up (see Figure 2). They then moved on to an introductory page for the first block where they were informed that they were to study 10 word pairs or face-name pairs (depending on the condition they were assigned to) for 4 s per item as well as an example of how this would later be tested. They were also informed that after studying, they would either be tested or get the chance to restudy the material and that this was decided at random in each block; in reality, this was determined by the condition to which they were assigned. Following this, they were exposed to either word pairs or face-name pairs for 4000 ms per item with a 500 ms interstimulus interval. After this, they were informed that the next page would consist of some simple math tasks that they would try to solve for a maximum of 30 s, after which they were automatically redirected to either test or restudy. After this distraction task, they were informed that they would either be tested on what they studied or would get to restudy it. During testing, they were exposed to the same word pairs/face-name-pairs that they were exposed to at the beginning of the block but were now only shown the Swahili word in the word pair or the face without the name, whereupon Swedish or English translation (depending on language version of the experiment) of the word pair and the name would be provided. During repetition, they received similar information as at the beginning of the block and were then presented with the exact same exposure again. After being tested or by restudying, the block was finished. This was repeated three times with negligible changes in the information (as a result of different types of exposures, for example) in the total of four blocks. After the test in the fourth block, everyone was given information that they would undergo a final test that consisted of all the parts previously studied (i.e., 10 word pairs and 30 face-name pairs, or 40 face-name pairs depending on the level of exposure material). After completing the cumulative test, they were asked to provide demographic information about their age and gender and were then thanked on a page that described the phenomenon being investigated and were provided contact information to the researchers.

### 2.5. Data Analysis

Regarding the scoring of the responses, close misspellings of words/names were considered correct (such as in [8] and [19]). For example, there were several misspellings on Evelina which were all scored as correct (“Eveline”, “Evelinq”, “Ewelina”). In a few isolated cases, a more liberal correction was used, even though the responses in question actually constituted some other closely related name than those sought (e.g., “Sanne” was considered correct for Sanna and “Sofie” correct for Sofia). This was considered reasonable as there were no other names similar to these and could therefore not be prior list intrusions. In some cases, when the spelling deviated slightly more or if there was some ambiguity about the interpretation of the response, stricter scoring was adopted for all instances of the same responses, which were then scored as incorrect. The scoring was carried out manually by one author but was reviewed together with another author afterwards to ensure compliance with the guidelines that had been decided upon before the scoring began.

Statistical analysis of the scores was carried out in IBM SPSS Statistics 26. The alpha level for all statistical tests was set to 0.05.

### 2.6. Ethical Aspects

This study was conducted online using PsyToolkit. This service collects IP addresses from the participants and stores them on their servers, but this information was not saved as part of the data collected in this study. All participation was completely anonymous, and the only data gathered from every participant was their gender and age.

On the introductory page, participants were informed that the experiment was completely anonymous, and that participation could be terminated at any time by simply leaving the study. The participants were informed that by starting the experiment they agreed to participate in the experiment.

Since all faces shown in the study were retrieved from https://thispersondoesnotexist.com (accessed on 1 June 2021), no existing person was shown as part of the experimental material.

## 3. Results

### 3.1. Interim Test in the Fourth Block

The number of correct responses as well as the number of prior list intrusions for the interim test in Block 4 are shown in Table 1. The Classic-Test group had, on average, more correctly recalled responses compared to the other groups. Both test groups (Transfer-Test and Classic-Test) produced the least number of prior list intrusions compared to the Initial Test groups as well as the Restudy groups. A clear difference can be observed between Test, Initial-Test, and Restudy in terms of prior list intrusions (regardless of Material) where the Test groups and Initial Test groups performed better than the Restudy groups.

No statistically significant interaction effect could be observed in a two-factor ANOVA between Test Occasions and Material on the number of correct responses in the fourth block’s test, F(2, 88) = 0.23, *p* = 0.79, η^2^ = 0.005. No main effect was observed on either Material, F(1, 88) = 0.17, *p* = 0.68, η^2^ = 0.002, or Test Occasions, F(2, 88) = 0.29, *p* = 0.75, η^2^ = 0.007. This data does not support the hypothesis that testing in every block or in an initial block would potentiate learning compared to repetition.

A two-factor ANOVA on the number of prior list intrusions between Test Occasions × Materials showed no statistically significant interaction effect, F(2, 88) = 0.51, *p* = 0.6, η^2^ = 0.012 but revealed a statistically significant main effect on the independent variable Test Occasions, F(2, 88) = 4.04, *p* = 0.021, η^2^ = 0.084 with a statistically significant mean difference between the test groups (M = 1.03, SD = 1.56) and the repetition groups (M = 2.42, SD = 2.39) at −1.39 in a number of prior list intrusions after a Tukey HSD post-hoc test, *p* = 0.014. This result indicates that proactive interference is reduced through interim testing.

Furthermore, a linear indication was observed in the mean values for prior list intrusions where the initially tested groups (M = 1.75, SD = 1.65) were between the means for the test and repetition groups. A Pearson’s r correlation test was performed post-hoc for the number of prior list intrusions for the Test, Initial test, and Restudy groups that showed a statistically significant, moderate correlation, r(92) = 0.29, *p* = 0.004. This result indicates that a test at the beginning of a study session can provide some protection against proactive interference in subsequent study blocks compared to restudying.

### 3.2. Cumulative Test

As word pairs have been shown to be easier to remember than face-name pairs [19], the items included in Block 1 were excluded from the analysis of the cumulative tests, regardless of whether they were face-name pairs or word pairs. Henceforth, where the results of the cumulative test are discussed, they will refer to this excluding definition where only Blocks 2, 3, and 4 are included in the analysis.

The number of correct responses for the cumulative test is shown in Table 2.

A two-factor ANOVA showed that there was no statistically significant interaction effect between Material and Test Occasions on the number of correct responses in the cumulative test, F(2, 88) = 0.1, *p* = 0.9, η^2^ = 0.002. There were also no statistically significant main effects on either Material, F(1, 88) = 0.21, *p* = 0.65, η^2^ = 0.002 or Test Occasions, F(2, 88) = 0.16, *p* = 0.85, η^2^ = 0.004. Thus, the data shows no statistically significant effect of forward testing regarding the cumulative test. The hypothesis that there would be some improvement in the results for the groups that received an initial test (regardless of Material) compared to the Restudy groups is not supported by this data.

## 4. Discussion

The purpose of this study was to further investigate the boundaries surrounding learning and interference within the paradigm for test potentiated learning by (1) attempting to reproduce the robust effect exhibited by forward testing on face-name pairs through an approach with potentially higher ecological validity by comparing testing with repetition, but also (2) to examine the effect of an initial test on the learning of subsequent information, as well as (3) to see whether Yang et al.’s [19] three-block-one-block transfer paradigm can be translated into a one-block-three-block transfer paradigm.

Despite the similarities in execution with which this study resembled the face-name pair studies [8,9,19] with the modification from distraction group to restudy group with the aim to increase the ecological validity of the study (in accordance with experiment 3 in [7]), no statistically significant effect on forward testing regarding the memory of faces and names could be shown on either the interim test on Block 4 or the final cumulative test. Since no effect of forward testing regarding memory of faces and names was shown, it is not possible to draw any conclusions about what effect the initial domain change could have on learning subsequent materials. On the other hand, a statistically significant reduction in the number of prior list intrusions was shown for the test groups compared with the repetition groups, which provides support for the theory of reducing proactive interference. An indication of reduced proactive interference in relation to the Restudy groups was also observed for the Initial Test groups, which was found to be statistically significant in a post-hoc correlation analysis. A possible explanation for this result could be that the interim tests caused mental context changes between the studied blocks, which made it easier for the test groups to distinguish the study blocks in the interim test on Block 4. This explanation is thus consistent with the theory of reduced proactive interference. According to this theory, interim testing makes it easier to distinguish separate study blocks from each other, which leads to decreased proactive interference. Since the Initial Test groups were tested only in Blocks 1 and 4, they should, according to the theory of reduced proactive interference, display more prior list intrusions from the blocks where they were not tested, which is in line with the results of this study (see Appendix A, Table A2).

Even though Szpunar et al. [7] found no difference in word recall between the distraction group and the restudy group, there could potentially be a difference between a distraction group and a restudy group when it comes to the recollection of face-name pairs. At fairly similar levels of exposure opportunities (as seen in the present study, where a second exposure opportunity was provided for all groups through either testing or restudying), a potential interpretation of the results in this study is that the type of materials used (face-name pairs) does not benefit from testing in comparison with repetition, in the short term. A replication of this study but with an additional group that would only be allowed to undergo one exposure per block with extra math between the blocks, similar to the previous studies that examined the recollection of face-name pairs without a restudy group [8,9,19] would need to be carried out in order to exclude this potential effect.

The results of this study allow some questionability regarding the robustness of FTE. Where previous studies have been able to show statistically significant results in favour of the phenomenon with almost exclusively large effect sizes, it is surprising that this study has not been able to show this effect, or even indications that the effect exists. As previously described regarding the potential negative effects that can occur with additional associations in an FTE paradigm, it is possible that yet unexplored aspects of the phenomenon could underlie this result. This sheds further light on the question of how specific the implementation of the phenomenon needs to be in order to potentiate learning.

### 4.1. Limitations

Since the scoring of responses has been done manually and is a relatively subjective method for interpreting correct and incorrect responses, we include this as a potential limitation. If a stricter or more liberal interpretation of the responses had been implemented, the result might have been somewhat different, even if this is perceived by us as an unlikely confounding factor. As previously described, where there was any ambiguity regarding the interpretation of the responses, a stricter scoring was adopted in favour of false negatives (i.e., more correct responses scored as incorrect) over false positives (i.e., more incorrect responses scored as correct) responses.

Considering that the study was conducted online, there are several potential limitations to mention regarding this. Given this fact, it has been difficult to control for cheating (although participants were initially asked not to use external aids), focus, and study environment, all three of which could potentially be the basis for the distribution in the results. However, studies comparing laboratory and online cognitive experiments have found that the online format does not generally seem to reduce data quality (see, e.g., [37]).

Since the sample may have an overrepresentation of persons who have previous knowledge of psychological effects, it is not inconceivable that this may have influenced the result. However, to our knowledge, no research has been done to assess whether knowledge of FTE prior to participation influences the result of forward testing.

As the distribution of correct responses was between 0 and 10 on the interim test on the fourth block and between 0 and 25 on the cumulative test (see Appendix A, Table A1, for descriptive statistics on the number of correct responses as well as prior list intrusions) regardless of condition, this indicates that more or less efficient memory techniques have been used. Since we did not control for the use of memory techniques, it cannot be ruled out that these may have disrupted a possible effect of forward testing. However, since no previous research has controlled for the usage of memory techniques, this was not accounted for in the design of this experiment. It should be added, however, that memory techniques should be controlled for by randomized assignment to the various conditions.

Another issue to consider is that the study combined data from a Swedish and an English version. Although we appreciate that this could potentially introduce more variation (i.e., noise) in the data, we concluded that it would not systematically influence any of the hypotheses and the increased power resulting from the larger sample size outweigh any potential drawbacks.

Because this study claims higher ecological validity by comparing the two study techniques testing and repetition (instead of distraction), it is retrospectively reasonable to question the choice of materials in terms of ecological validity. Studying face-name pairs is most likely unusual in educational as well as in professional contexts and a choice of study material that would have better reflected more common educational content would undoubtedly have increased the ecological validity when it comes to the potential practical implications of this study.

A potential methodological limitation of this study is that the focus has been on two independent variables (Materials and Testing Occasions) which are not individually explored exhaustively in the present study. It is possible that the result would have been reflected differently by focusing on one of these two independent variables while omitting the other. Suggested approaches for both of those options are presented in Section Future research.

### 4.2. Future Research

Future studies with a similar design to the present study are recommended to go even deeper into the area of the transferability of the forward testing effect and the extent to which this transferability is useful. Instead of having only an initial block of domain-separate material, an investigation of what effect transferability has on several different combinations of domain-separate materials would be needed. An example could be to compare the effect between test groups only, where Blocks 1, 2, and 3 have either the first and second, or the second and third block as domain-separate material where the material in Block 4 is kept constant. This could be done to see what effect this type of domain change has on the learning of the last block.

Although the present study used the theory of test expectancy in the design of the groups that received an initial test, it was never investigated to what extent the expectation to be tested was affected by this initial block compared to the repetition groups. This is something that could be investigated further by letting the participants state their test expectancy before each block, such as in [8], for example. This would provide clues as to what effect an initial test has on subsequent test expectations and thus provide further indications of whether the theory of test expectancy is an underlying mechanism for the effect of forward testing or not.

Although we include study environment as a potential limitation in this study, we also want to highlight the potential for examining how forward testing is potentiated in different types of environments. Since, at the time of writing, the global situation during the SARS-CoV-2 pandemic to a very small extent allows for more distance education for students in many higher education institutions, it is more relevant than ever to examine what effect the choice of study environment has on learning. It is reasonable to assume that even before the pandemic, students had, to different extents, conduct self-studying (reading or similar activities) in places that are suboptimal for learning, but the effect of choice of environment and its various potential distractions is perhaps more relevant now than ever before.

## 5. Conclusions

Streamlining learning is as relevant today as ever and many different study techniques have been advocated. One of the most effective study techniques has shown to be testing, and this study has built on the phenomenon within testing that has come to be test-potentiated learning of new materials, the forward testing effect. Although the phenomenon is relatively young, many studies have been able to show a robust effect on learning many different types of materials. However, the phenomenon is still in need of more research, which is made clear by the different theories that have been proposed to form the underlying mechanisms for the effect of forward testing as well as the scattered results that history has been able to show with everything from clearly positive effects to potentially negative effects on recollection through subsequently added associations to, finally, the results of this study, which failed to show any beneficial effect on learning new materials through testing in relation to repetition. The present study does, however, reveal a clear effect on the build-up of proactive interference where testing reduces this on a level that is statistically significant when compared to repetition.

Given these contraindicative results, carrying out practical implementations of the phenomenon in order to promote learning should be done with caution. However, interim testing could be recommended in cases where similar lists or objects are to be studied to avoid incorrect associations in accordance with the theory of reduced build-up of proactive interference.

## Figures and Tables

**Table 1:** Means and standard deviations over the number of correct responses and the number of prior list intrusions in the fourth block’s interim test.

	Correct Responses		Prior List Intrusions	
Test Occasions	M	SD	M	SD
Transfer-Test	2.56	1.59	0.63	1.36
Transfer-Initial Test	2.44	2.19	1.81	1.83
Transfer-Restudy	2.87	2.72	2.27	2.69
In total	2.62	2.16	1.55	2.09
Classic-Test	3.2	2.65	1.47	1.69
Classic-Initial Test	2.5	2.34	1.69	1.49
Classic-Restudy	2.75	2.08	2.56	2.07
In total	2.81	2.33	1.91	1.79
Test (Transfer + Classic)	2.87	2.16	1.03	1.56
Initial Test (Transfer + Classic)	2.47	2.23	1.75	1.65
Restudy (Transfer + Classic)	2.81	2.37	2.42	2.35
In total	2.71	2.24	1.73	1.95

**Table 2:** Means and standard deviations over the number of correct responses in the cumulative test.

Test Occasions	M	SD
Transfer-Test	7.19	4.71
Transfer-Initial Test	5.81	5.37
Transfer-Restudy	6.8	5.91
In total	6.6	5.25
Classic-Test	7.27	5.65
Classic-Initial Test	7.06	6.28
Classic-Restudy	7.06	5.41
In total	7.13	5.67
Test (Transfer + Classic)	7.23	5.1
Initial Test (Transfer + Classic)	6.44	5.78
Restudy (Transfer + Classic)	6.94	5.56
In total	6.86	5.44

## Data Availability

The data presented in this study are openly available in FigShare at https://doi.org/10.6084/m9.figshare.14915559 (accessed on 6 July 2021).

## Appendix A

**Table A1:** Number of correct responses and prior list intrusions.

	N	Minimum	Maximum	M	SD
Block 4, number of correct responses	94	0	10	2.71	2.24
Block 4, prior list intrusions	94	0	9	1.73	1.95
Cumulative test, number of correct responses	94	0	25	6.86	5.44

**Table A2:** M and SD over the number of list intrusions from the previously occurring lists, respectively, in the fourth block’s interim test.

	List Intrusions from List 1		List Intrusions from List 2		List Intrusions from List 3	
	M	SD	M	SD	M	SD
Classic-Test	0.40	0.74	0.47	0.64	0.60	0.99
Classic-Initial Test	0.19	0.40	0.81	0.75	0.75	1.00
Classic-Restudy	0.88	1.02	0.50	0.73	1.19	1.28
Total	0.49	0.80	0.60	0.71	0.85	1.10
